# Effect of tiotropium on night-time awakening and daily rescue medication use in patients with COPD

**DOI:** 10.1186/s12931-016-0340-9

**Published:** 2016-03-12

**Authors:** Peter M. A. Calverley, Stephen I. Rennard, Emmanuelle Clerisme-Beaty, Norbert Metzdorf, Valentina Bayer Zubek, Richard ZuWallack

**Affiliations:** Clinical Science Centre, University Hospital Aintree, Longmoor Lane, Liverpool, L9 7AL UK; University of Nebraska Medical Center, Omaha, NE USA; Boehringer Ingelheim Pharmaceuticals Inc, Ridgefield, CT USA; Boehringer Ingelheim GmbH, Ingelheim, Germany; St Francis Hospital Medical Center, Hartford, CT USA

**Keywords:** Albuterol, Bronchodilation, COPD, Long-acting anticholinergic, Night-time awakening, Peak expiratory flow rate, Rescue medication, Tiotropium

## Abstract

**Background:**

Several small studies found night-time awakenings due to COPD symptoms were associated with decreased health status. In this study, night-time awakenings in patients with COPD were examined and effects of tiotropium therapy evaluated.

**Methods:**

This study was a post hoc, exploratory, pooled analysis of twin, multicenter, double-blind, randomized, placebo-controlled, parallel-group trials. Patients with stable moderate-to-severe COPD were randomized to tiotropium HandiHaler® (*n* = 550) or placebo (*n* = 371) and followed for 13 weeks. During a 2-week, pre-treatment baseline period and for 13 weeks on treatment, self-reported night-time awakenings due to COPD symptoms, rescue medication (albuterol) use, and morning and evening peak expiratory flow rate (PEFR) were recorded daily. Nightly, COPD-related awakenings were scored: 0 = no awakenings; 1 = 1 awakening; 2 = 2–3 awakenings; or 3 = awake most of the night. Health-related quality-of-life (HRQoL) and energy–fatigue questionnaires were completed at baseline and during treatment.

**Results:**

Patients were aged 65.2 ± 8.7 years (mean ± SD), with a mean pre-bronchodilator FEV_1_ of 36.1 ± 13.5 % predicted normal at baseline. Data for night-time awakenings and albuterol use were available for 543 (99 %) patients on tiotropium and 352 (95 %) on placebo. At baseline, 280 (51.5 %) patients on tiotropium and 179 (50.1 %) on placebo reported ≥1 COPD-related night-time awakening per week. Over the 13-weeks’ treatment, tiotropium was associated with fewer night-time awakenings, with mean ± SE overall awakening scores per week of 0.356 ± 0.006 compared with 0.421 ± 0.007 for placebo (*p* < 0.001); means were significantly lower for tiotropium versus placebo in patients with baseline awakenings (*p* < 0.001), but not for those without baseline awakenings. COPD-related night-time awakenings were associated with increased nocturnal rescue medication use and lower HRQoL ratings in both treatment arms. Following start of treatment, tiotropium decreased patients’ use of rescue medication compared with placebo, and morning and evening adjusted means for PEFR were higher for tiotropium compared with placebo.

**Conclusions:**

Tiotropium is associated with decreased COPD-related night-time awakenings. Night-time awakenings are associated with increased nocturnal rescue medication use and may be a surrogate marker of symptom control in patients with COPD.

**Electronic supplementary material:**

The online version of this article (doi:10.1186/s12931-016-0340-9) contains supplementary material, which is available to authorized users.

## Background

The onset of sleep poses particular problems for the respiratory system. There is a well-documented circadian rhythm in lung function, with forced expiratory volume in 1 s (FEV_1_) falling by approximately 150 mL overnight in healthy subjects [1]. This change is enhanced in patients with asthma, who often complain of nocturnal respiratory symptoms [2], whereas, patients with chronic obstructive pulmonary disease (COPD) show falls in overnight FEV_1_ similar to those seen in healthy individuals [3]. Although sleep quality is known to be poor in COPD [4, 5], increased nocturnal symptoms in this population have only been demonstrated recently [6], and night-time awakening could be considered a potential surrogate for poor disease control.

The relationship of night-time and morning symptoms in COPD to changes in lung function is unclear, since relatively little attention has been paid to recording the time of day when symptoms are most prevalent. In patients with COPD, McNicholas et al. [7] found that the long-acting anti-muscarinic drug tiotropium significantly reduced the degree of rapid-eye-movement sleep-related desaturation and improved post-sleep FEV_1_ compared with placebo. In comparison with tiotropium alone, the addition of an inhaled corticosteroid/long-acting beta-agonist (LABA) combination to tiotropium, was associated with fewer morning symptoms in patients treated with this more intensive regime [8]. However, to date, there are relatively few data available on the impact of once-daily long-acting inhaled bronchodilators on night-time symptoms, either as reported by patients, or reflected in the use of rescue medication to control respiratory symptoms at night or in the morning.

We hypothesized that the use of tiotropium would be associated with less nocturnal awakening due to respiratory symptoms and less rescue medication use at night and during the first half of the waking day. To test these hypotheses, we conducted a post hoc analysis of two identically-designed, randomized, placebo-controlled clinical trials in which night-time awakening and rescue medication data were available. In addition, the effect of night-time awakenings on nocturnal rescue medication use and health status were also evaluated to assess further the clinical impact of these events.

## Methods

### Study design

This post hoc exploratory study was a pooled analysis of twin, multicenter, double-blind, randomized, placebo-controlled, parallel-group trials, conducted at 50 sites in the United States. The trials evaluated the efficacy and safety of tiotropium delivered by a dry powder inhaler in patients with COPD. Each trial had three phases, a 2-week baseline period, an initial 13-week safety and efficacy treatment phase, and an extension phase to Week 49 to collect data on long-term lung function and exacerbations. During the treatment phase, data on night-time awakenings due to COPD symptoms were collected over the first 13 weeks only and not during the extension phase. Efficacy and safety results for the whole duration of the trials have been reported previously [9].

The primary efficacy endpoint in the original trials was trough FEV_1_ change from baseline after 13 weeks of treatment. Pre-specified secondary endpoints included in the analyses were rescue medication use, morning and evening PEFR, health-related quality-of-life (HRQoL) measures, and energy and fatigue scores. These secondary endpoints, along with scores for night-time awakenings due to COPD symptoms, were collected from patient diaries and questionnaires. This is a retrospective study, which reports post hoc results from twin multicenter studies. In the originating twin studies, the study protocol was approved by the Institutional Review Board at each center. The IRB used most frequently was the Western Institutional Review Board, 3535 7th Ave. SW, Olympian WA 98502, USA. A full list of all IRBs could be provided if this is necessary.

All patients gave written informed consent and the study protocol was approved by the Institutional Review Board at each center.

### Study participants

Study participants were outpatients, aged ≥40 years, with a >10 pack-years smoking history, and a clinical diagnosis of COPD as defined by the American Thoracic Society [10]. Patients had stable, moderate-to-severe airway obstruction, with a pre-bronchodilator FEV_1_ of ≤65 % of predicted normal according to Morris [11] and a FEV_1_/forced vital capacity (FVC) of ≤70 %.

Patients were excluded if they had significant disease other than COPD that might influence the results of the study or put them at risk if they participated. Other exclusion criteria included: a history of asthma, allergic rhinitis, or atopy; total blood eosinophilia ≥600/mm^3^; and regular use of daytime oxygen therapy. Patients with a history of heart failure within 3 years, myocardial infarction within 1 year, or cardiac arrhythmia treated with drugs were also excluded.

During the study, patients were allowed most concomitant medications, with the following restrictions on respiratory medications. Inhaled anticholinergic drugs (e.g., ipratropium bromide and atropine) were allowed during the baseline period but, with the exception of the study drug, not during the treatment period. Albuterol rescue medication was allowed as needed throughout the study. Theophylline preparations (excluding 24-h preparations), inhaled corticosteroids, and doses of oral corticosteroids (equivalent to ≤10 mg prednisone daily or ≤20 mg every other day) were allowed if at a stable dose for ≥6 weeks prior to screening and throughout the study period. For acute exacerbations, any additional medications, including antibiotics, were permitted as required. However, patients were allowed only two, 7-day increases in the dose or the addition of oral steroids or theophylline. Other investigational drugs, all beta blockers, cromolyn sodium, nedocromil sodium, oral beta-adrenergics, or LABAs were not allowed for 1 month prior to the baseline period or throughout the study period. Patients were excluded if they were taking antihistamines (H_1_ receptor antagonists) at screening. Patients not meeting the concomitant medication requirements were excluded.

### Study procedures

#### Screening and baseline period

At a screening visit, demographic and medical history data were collected; a 12-lead ECG, physical examination, pulmonary function testing, laboratory evaluations, and measurement of serum theophylline levels (if used) were also performed. Spirometry was conducted to American Thoracic Society standards [12], and % of predicted normal values were calculated using Morris et al. [11] and the European Community for Steel and Coal (ECSC) [13] equations. Qualifying patients entered a 2-week baseline period, during which time night-time awakenings, morning and evening PEFR, and albuterol rescue medication were recorded daily in patient diaries. The baseline week is defined as Week 0, the week immediately preceding the date of first treatment.

#### Treatments

After the baseline period, patients were randomized in a 3:2 ratio to tiotropium (18 μg/day tiotropium; Spiriva®; Boehringer Ingelheim) or matching placebo, taken once daily at the same time each morning (between 8.00 am and 10.00 am) from a dry powder inhaler (HandiHaler®; Boehringer Ingelheim, Ingelheim am Rhein, Germany).

#### Night-time awakening scores

Night-time awakenings due to COPD symptoms (wheezing, shortness of breath, and coughing) were scored for each night in the baseline week (Week 0) before treatment started and for 13 weeks post-randomization. For each night, patients were asked to score their COPD-related awakenings using the following scale: 0 for no awakenings; 1 for one awakening; 2 for two or three awakenings; and 3 for being awake for most of the night. Because of the variability in night-time awakenings over time, a weekly mean awakening score was calculated for each patient and averaged over 13 weeks. For any given week, if a patient did not experience any night-time awakenings due to COPD symptoms his/her weekly mean score was listed as 0 for that week. For example, for a patient who scored 0 for 3 nights, 1 for 2 nights and 2 for 2 nights, the weekly mean score was calculated as 6/7 = 0.86.

For subgroup analyses, patients were divided into those with and those without night-time awakening at baseline.

#### Rescue medication use

Rescue medication use, measured by the number of doses (consisting of one or two puffs) of rescue albuterol taken over 6-h intervals (6 am to noon, noon to 6 pm, 6 pm to midnight, and midnight to 6 am), was recorded by patients in their daily diaries. Rescue medication use was recorded both at baseline and during the 13-week treatment period. This was used to calculate the number of doses taken over each 24-h period. For each patient, weekly means were calculated for each of the four 6-h periods and for 24 h.

#### Peak expiratory flow rates

Patients recorded their peak expiratory flow rate (PEFR) measurements twice daily during the baseline and treatment periods using an AirWatch™ Monitor (Enact Health Management Systems, Mountain View, CA, USA). Morning PEFR was measured by patients immediately upon rising before any medication use. Evening PEFR was measured before bedtime. Measurements were recorded in daily diaries and weekly means were calculated for each patient.

#### Questionnaires

To assess general HRQoL, the generic, 36-item Short-Form Health Survey (SF-36®) [14] was administered prior to randomization and at 13 weeks from start of treatment. The SF-36 scales were scored separately and transformed to a scale of 0 to 100, where 0 indicates the worst and 100 the best HRQoL.

At the end of the baseline period, at the end of Week 1, and every 3 weeks thereafter until Week 13, patients were asked three questions related to the perception of their energy, fatigue, and severity of their respiratory condition (only baseline and Week 13 data are reported here). Energy was scored from 1 = very good to 5 = very poor, fatigue was scored from 1 = very severe to 6 = no fatigue, and severity of the respiratory condition was scored from 1 = very severe to 6 = no problems at all (see Additional file 1: Table S1 for full details).

### Statistical analyses

Since all statistical analyses were post hoc and exploratory, all p-values can only be interpreted as nominal values. Analyses used the full analysis set, which included all treated patients who had baseline data and data for 2 weeks on treatment. Baseline was defined as the week before treatment, and baseline values were calculated for each treatment, as opposed to a common mean across the treatments. For each patient, weekly means for night-time awakening score, rescue medication use, and PEFR were calculated from the daily diary records, and the mean ± standard error (SE) of the weekly means were calculated and compared across the treatment groups. Last observation carried forward was used for data imputation. For each patient, the “overall mean” for Weeks 1–13 was calculated as the average of his/her weekly means.

Analysis of covariance with treatment, baseline, and pooled centers as covariates was used as the statistical model. Adjusted means were estimated weekly and overall (Weeks 1–13) for night-time awakening, rescue medication, and morning and evening PEFR. Adjusted means for SF-36 and energy–fatigue questionnaire scores were calculated at the pre-specified key time points.

Pearson’s correlation was used to test associations between variables. To determine the role of night-time awakening as a marker of disease control, correlations between night-time awakening and each of the following variables were calculated using the patients’ weekly means: rescue medication, SF-36, and energy–fatigue.

For subgroup analyses, patients were split within each treatment group into two groups: (i) patients who reported at least one night when they had at least one awakening due to COPD symptoms at baseline (i.e. night-time awakening score >0) and (ii) patients without a single night-time awakening due to COPD symptoms at baseline (i.e. night-time awakening score = 0).

## Results

### Baseline demographics and clinical characteristics

Of 1287 patients screened, 550 patients were randomized to tiotropium and 371 to placebo. The first patient was enrolled into the trials on the 7 January 1997 and the last patient completed the 13-week phase on 30 September 1997. Of the patients with baseline data, one randomized to tiotropium and five to placebo were not included in the analyses because they had insufficient data for 2 weeks on treatment.

Patients were predominantly Caucasian, 65 % were male, with a mean ± SD age of 65.2 ± 8.7 years, smoking history of 61.2 ± 30.6 pack-years, and FEV_1_ % predicted of 36.1 ± 13.5 % (ECSC equation [13]). Baseline demographics and clinical characteristics were similar between the tiotropium and placebo treatment groups, and between the subgroups of patients with and without night-time awakening at baseline (Table 1).

**Table 1:** Baseline demographics and clinical characteristics of trial participants

	All patients		Night-time awakening at baseline		No night-time awakening at baseline	
	Tiotropium	Placebo	Tiotropium	Placebo	Tiotropium	Placebo
	(*n* = 544)	(*n* = 357)	(*n* = 280)	(*n* = 179)	(*n* = 264)	(*n* = 178)
Age, years	65.11 ± 8.50	65.30 ± 8.84	63.86 ± 8.68	63.56 ± 8.86	66.44 ± 8.12	67.05 ± 8.50
Male, n (%)	363 (66.7)	227 (63.6)	186 (66.4)	115 (64.2)	177 (67.0)	112 (62.9)
Race, n (%)						
White	518 (95.2)	330 (92.4)	263 (93.9)	159 (88.8)	255 (96.6)	171 (96.1)
Black	26 (4.8)	25 (7.0)	17 (6.1)	19 (10.6)	9 (3.4)	6 (3.4)
Asian	0 (0.0)	2 (0.6)	0 (0.0)	1 (0.6)	0 (0.0)	1 (0.6)
Body mass index, kg/m^2^	28.32 ± 5.66	28.07 ± 5.96	28.32 ± 5.58	27.59 ± 6.00	28.32 ± 5.77	28.55 ± 5.89
Alcohol use, n (%)						
Non-drinker	284 (52.2)	174 (48.7)	152 (54.3)	89 (49.7)	132 (50.0)	85 (47.8)
Average consumption	259 (47.6)	181 (50.7)	128 (45.7)	89 (49.7)	131 (49.6)	92 (51.7)
Excessive consumption	1 (0.2)	2 (0.6)	0 (0.0)	1 (0.6)	1 (0.4)	1 (0.6)
Smoking history, pack-years	62.59 ± 30.68	59.21 ± 30.53	64.31 ± 32.31	59.25 ± 31.41	60.77 ± 28.80	59.17 ± 29.72
Duration of COPD, years	8.64 ± 7.42	8.05 ± 6.70	8.88 ± 7.56	8.64 ± 7.42	8.38 ± 7.28	7.47 ± 5.84
Rescue medication use/24 h	3.66 ± 2.71	3.44 ± 2.57	4.30 ± 2.92	4.08 ± 2.77	2.99 ± 2.28	2.80 ± 2.18
FEV_1_, L	1.05 ± 0.41	1.01 ± 0.44	1.05 ± 0.42	1.00 ± 0.42	1.05 ± 0.41	1.02 ± 0.46
FEV_1_, % predicted (Morris equation [11])	38.02 ± 14.07	37.44 ± 14.18	36.88 ± 13.75	36.36 ± 13.91	39.22 ± 14.32	38.53 ± 14.41
FEV_1_, % predicted (ECSC equation [13])	36.29 ± 13.48	35.74 ± 13.62	35.22 ± 13.20	34.75 ± 13.38	37.43 ± 13.71	36.75 ± 13.82
FVC, L	2.31 ± 0.79	2.23 ± 0.78	2.28 ± 0.79	2.22 ± 0.78	2.35 ± 0.79	2.25 ± 0.78
FEV_1_/FVC, %	45.96 ± 11.60	45.51 ± 11.70	46.59 ± 11.52	45.52 ± 11.93	45.30 ± 11.67	45.51 ± 11.49

Data are mean ± standard deviation or number of patients (%)

COPD, chronic obstructive pulmonary disease; ECSC, European Community for Steel and Coal; FEV_1_, forced expiratory volume in 1 s; FVC, forced vital capacity

Data on rescue medication use and night-time awakening were available for 901 patients at baseline, with 51.5 % in the tiotropium and 50.1 % in the placebo groups reporting at least one night-time awakening per week.

### Night-time awakening scores

**Fig. 1:**
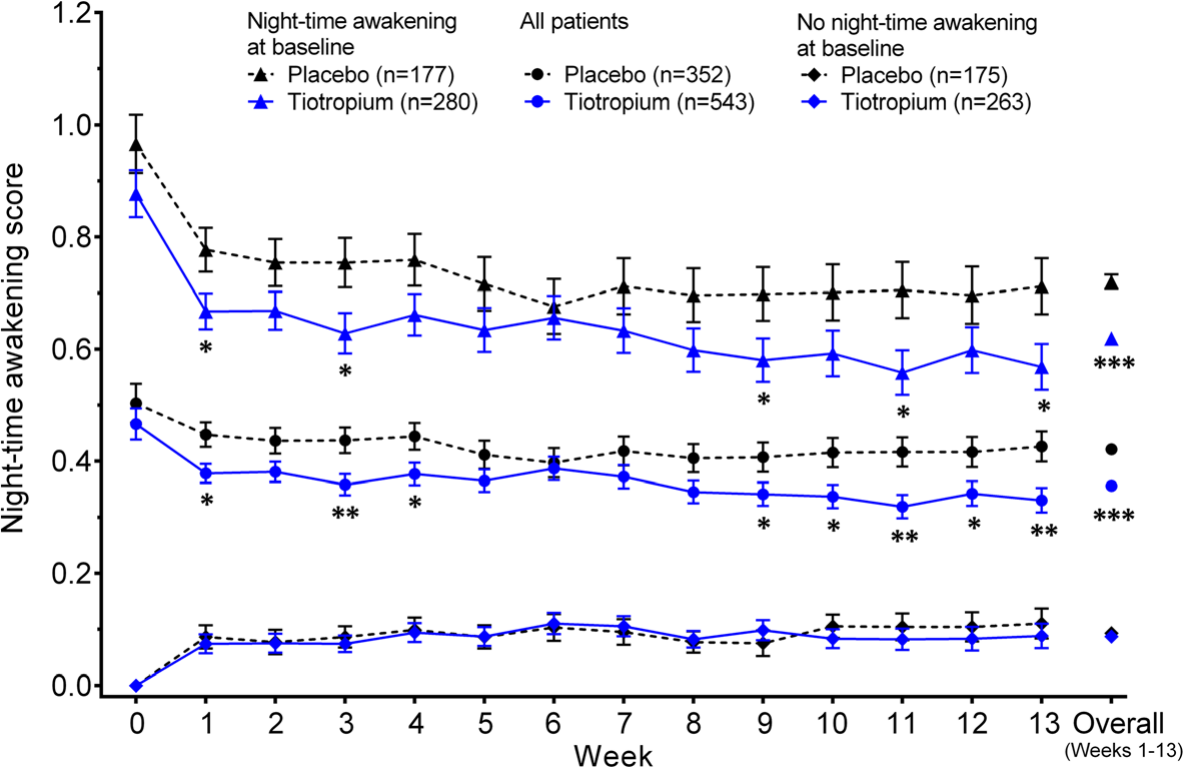
Adjusted weekly mean night-time awakening scores per night due to COPD symptoms. Data presented are for all patients and for the subgroups of patients who did and did not experience at least one night-time awakening due to COPD symptoms at baseline. Night-time awakening scores were based on the following: 0 for no awakenings; 1 for one awakening; 2 for two to three awakenings; and 3 for being awake for most of the night. Data are mean ± standard error. The means are adjusted for center effects and baseline. The overall mean is the average over the 13 on-treatment weekly means. There were no significant differences between tiotropium and placebo for the group of patients with no awakening at baseline at any time point. *p < 0.05; **p < 0.01; ***p < 0.001 for tiotropium versus placebo

Night-time awakening scores per night for all patients and for the subgroups with and without baseline night-time awakenings are shown in Fig. 1.

At baseline, adjusted treatment means, calculated from patients’ weekly means, for night-time awakening score per night were similar for the tiotropium and placebo treatment groups (0.466 ± 0.028 and 0.503 ± 0.035, respectively; *p* > 0.05). At Week 13, adjusted treatment means were significantly lower for tiotropium compared with placebo (0.330 ± 0.022 and 0.426 ± 0.027, respectively; *p* < 0.01). Adjusted overall treatment means (i.e. averaged over the entire 13-week treatment period) were also lower for tiotropium compared with placebo (0.356 ± 0.006 and 0.421 ± 0.007, respectively; *p* < 0.001).

**Fig. 2:**
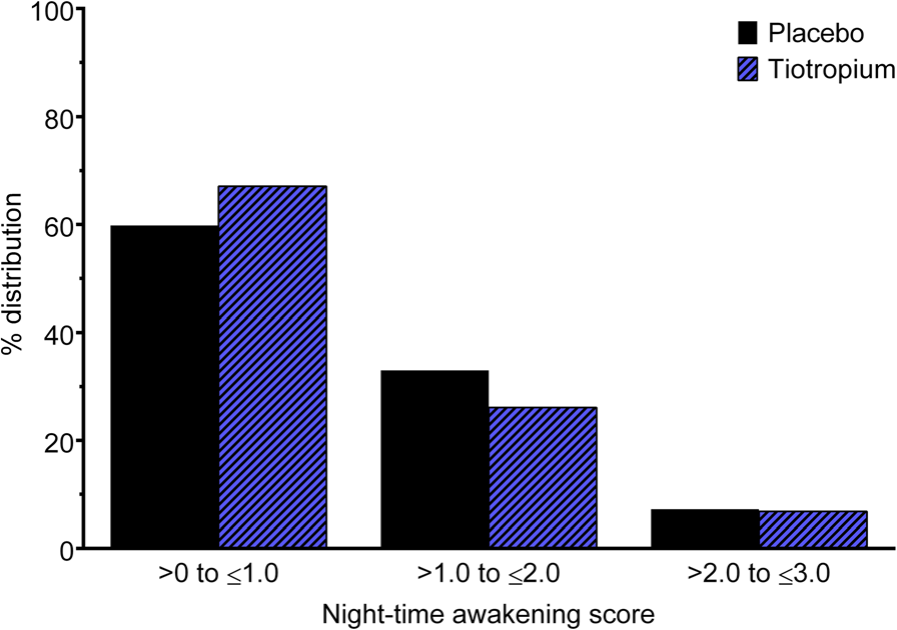
Baseline distribution of weekly mean scores of night-time awakening for patients who experienced at least one night-time awakening. For each night, patients scored: 0 for no awakenings; 1 for one awakening; 2 for two or three awakenings; and 3 for being awake for most of the night; these scores were used to calculate a weekly mean for each patient

In the subgroup analysis of patients with baseline night-time awakening, adjusted treatment means at baseline were similar for tiotropium and placebo (0.877 ± 0.042 and 0.966 ± 0.052, respectively; *p* > 0.05), as was the distribution of night-time awakening scores (Fig. 2). Adjusted treatment means were significantly lower for tiotropium compared with placebo at Week 13 (0.568 ± 0.041 and 0.712 ± 0.050, respectively; *p* < 0.05) and also when averaged over the entire 13-week treatment period (0.619 ± 0.100 and 0.720 ± 0.013, respectively; *p* < 0.001).

In the subgroup of patients without baseline night-time awakenings, the adjusted treatment means were not significantly different between the tiotropium and placebo groups at Week 13 (0.088 ± 0.022 and 0.110 ± 0.027, respectively; *p* > 0.05) or when averaged over the entire 13-week treatment period (0.087 ± 0.005 and 0.093 ± 0.006, respectively; *p* > 0.05).

### Rescue medication use

At baseline, the mean number of rescue medication doses per 24-h time period was similar for the tiotropium and placebo treatment groups (mean ± SD, 3.66 ± 2.71 and 3.44 ± 2.57, respectively). Following the start of treatment, tiotropium decreased patients’ use of rescue medication compared with placebo (see Additional file 2: Figure S1).

**Fig. 3:**
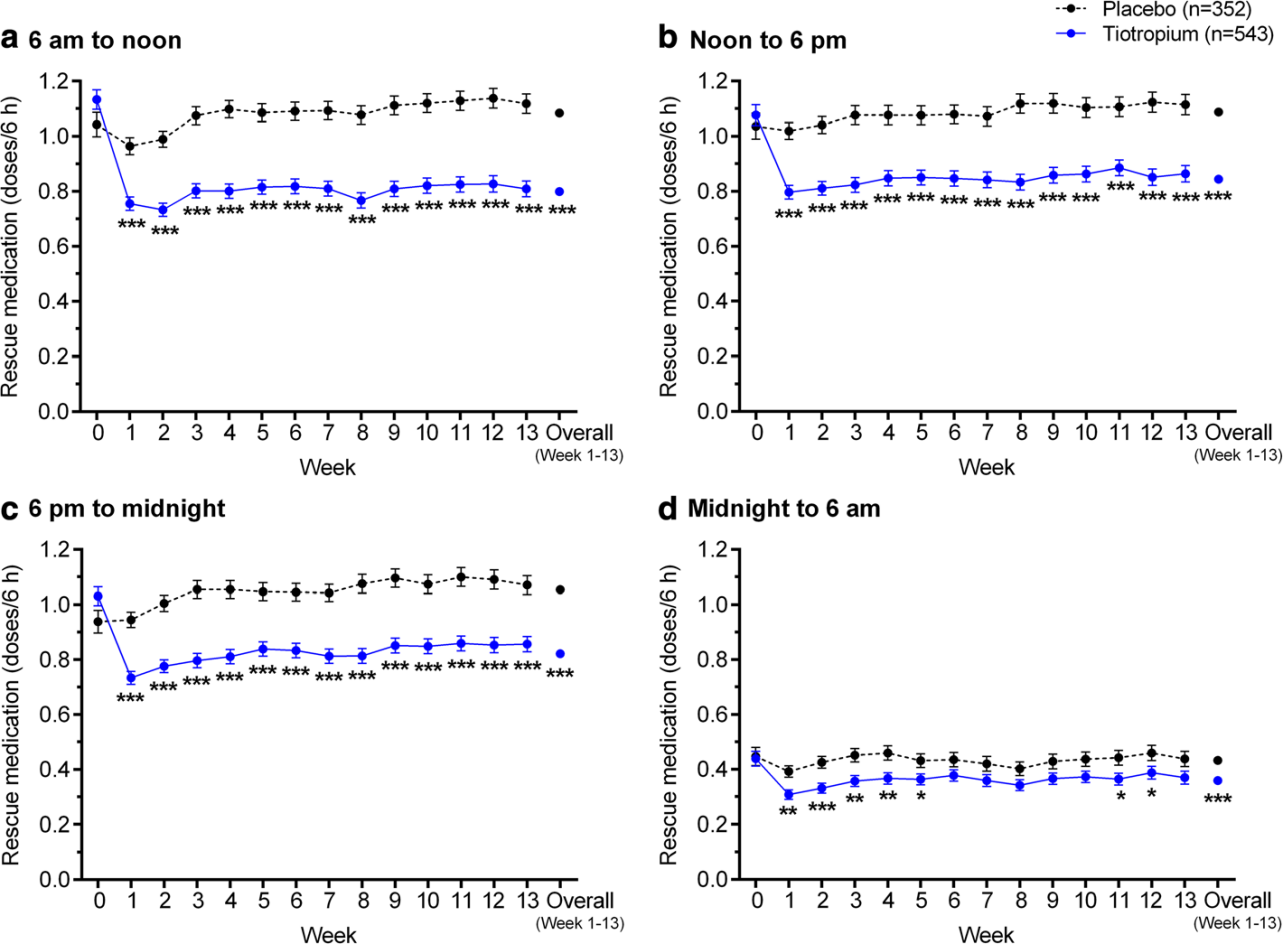
Adjusted weekly mean rescue medication doses/6-h periods; **a** 6 am to noon, **b** noon to 6 pm, **c** 6 pm to midnight, and **d** midnight to 6 am. A dose of rescue medication use was defined as one or two puffs of albuterol. Data are mean ± standard error. The means are adjusted for center effects and baseline. The overall mean is the average over the 13 weekly means. *p < 0.05; **p < 0.01; ***p < 0.001 for tiotropium versus placebo

The proportions of patients with rescue medication use at baseline by 6-h time periods were: 84.2 % (759 patients) from 6 am to noon; 80.5 % (725 patients) from noon to 6 pm; 80.9 % (729 patients) from 6 pm to midnight; and 50.9 % (459/901 patients) from midnight to 6 am;. Weekly mean number of rescue medication doses per 6-h time periods are shown in Fig. 3a–d. During each of the 6-h time periods, rescue medication use was consistently lower for tiotropium compared with placebo following start of treatment. Throughout the baseline and treatment periods, rescue medication use was lowest in the midnight to 6 am period for both the placebo and tiotropium treatment groups (Fig. 3d).

For both the tiotropium and placebo treatment groups, weekly mean rescue medication doses taken during the night-time (6 pm to 6 am) and daytime (6 am to 6 pm) were strongly correlated both at baseline and throughout the treatment period (baseline: *r* = 0.78 for tiotropium and *r* = 0.77 for placebo; Weeks 1–13: range *r* = 0.76–0.81 for tiotropium and *r* = 0.70–0.80 for placebo; and overall [Weeks 1–13]: *r* = 0.84 for tiotropium and *r* = 0.78 for placebo; *p* < 0.001 for all correlations).

### Correlation between rescue medication use and night-time awakenings

Rescue medication use and night-time awakening score were positively correlated for both the tiotropium and placebo treatment groups over all time periods (*p* < 0.001; Table 2). The strongest correlation was between rescue medication use from midnight to 6 am and night-time awakening score (overall [Weeks 1–13]: *r* = 0.55 for tiotropium and *r* = 0.61 for placebo; both *p* < 0.001).

**Table 2:** Pearson’s correlation coefficients: weekly mean rescue medication doses and weekly mean night-time awakening scores

Rescue medication correlation with night-time awakening	Tiotropium		Placebo	
	*n*	*r*	*n*	*r*
24 h				
Baseline	544	0.30***	357	0.34***
Overall (Weeks 1–13)	543	0.41***	352	0.39***
6 pm to 6 am				
Baseline	544	0.39***	357	0.40***
Overall (Weeks 1–13)	543	0.48***	352	0.51***
6 pm to midnight (12 am)				
Baseline	544	0.24***	357	0.26***
Overall (Weeks 1–13)	543	0.34***	352	0.31***
Midnight (12 am) to 6 am				
Baseline	544	0.47***	357	0.43***
Overall (Weeks 1–13)	543	0.55***	352	0.61***
6 am to 6 pm				
Baseline	544	0.20***	357	0.25***
Overall (Weeks 1–13)	543	0.32***	352	0.26***

***p < 0.001

### Peak expiratory flow rates

**Fig. 4:**
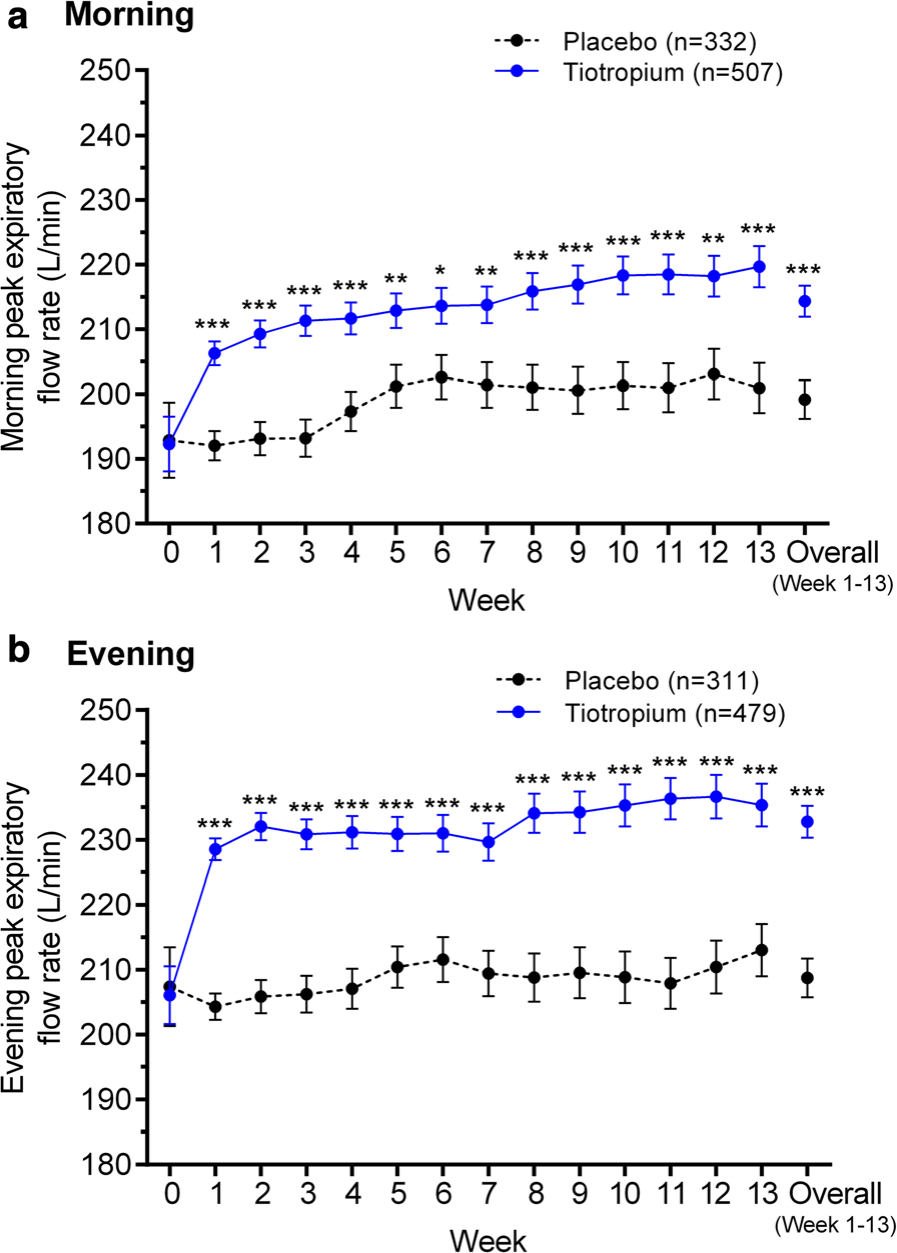
Adjusted weekly mean **a** morning and **b** evening peak expiratory flow rates. Data are mean ± standard error. The means are adjusted for center effects and baseline. The overall mean is the average over the 13 weekly means for tiotropium versus placebo. *p < 0.05; **p < 0.01; ***p < 0.001 for tiotropium versus placebo

The weekly means for morning PEFR at baseline were 192.3 ± 4.2 L/min for the tiotropium and 192.9 ± 5.8 L/min for the placebo treatment groups (Fig. 4a) and for evening PEFR were 206.1 ± 4.5 L/min for tiotropium and 207.4 ± 6.1 L/min for placebo (Fig. 4b). For all treatment weeks, both morning and evening adjusted means for PEFR were higher for tiotropium compared with placebo (all *p* < 0.05; Fig. 4a and b, respectively).

### Questionnaires

After 13 weeks of treatment (Day 92), patients in the tiotropium treatment group had greater (i.e., more favorable) SF-36 scores for Physical Function, Role Physical, General Physical Health, and Physical Health Summary compared with patients receiving placebo (all *p* < 0.01; Additional file 3: Figure S2A). In a subgroup analysis at Week 13, patients without baseline night-time awakening had higher scores for Physical Function, Physical Health Summary, and Role Emotional in the tiotropium group compared with placebo (*p* < 0.05; Additional file 3: Figure S2B) and patients with baseline night-time awakening had higher scores for General Physical Health and Physical Health Summary in the tiotropium group compared with placebo (*p* < 0.01 and *p* < 0.05, respectively; Additional file 3: Figure S2C). Patients without baseline night-time awakening had a larger treatment effect for Role Physical, Bodily Pain, Role Emotional, and General Mental Health compared with those with baseline night-time awakening, conversely, patients with baseline night-time awakening had a larger treatment effect for Vitality.

In both the tiotropium and placebo treatment groups, there were negative and broadly consistent correlations at baseline and Week 13 between night-time awakening scores and each of the SF-36 domains (all *p* < 0.001; see Additional file 4: Table S2). For tiotropium, correlation coefficients were in the ranges: *r* = −0.297 to −0.216 at baseline and *r* = −0.233 to −0.175 at Week 13, and for placebo: *r* = −0.326 to −0.213 at baseline and *r* = −0.380 to −0.208 at Week 13. Correlations between the overall mean for Weeks 1–13 for night-time awakening scores and SF-36 domain scores at Week 13 were also investigated and gave broadly similar results to those seen for the Week 13 comparisons (all *p* < 0.001; see Additional file 4: Table S2).

Results for the energy, fatigue, and severity of condition questionnaire are presented in the Additional file 5: Tables S3 and S4.

## Discussion

This analysis has shown that night-time awakenings due to COPD symptoms were present at baseline in around half of the patients studied. Tiotropium treatment was associated with decreased night-time awakenings. These awakenings were correlated with the use of rescue medication and, to a lesser degree, impairment of health status and increased fatigue, suggesting that night-time awakening could be a potential surrogate for poor disease control.

Although considerable effort has been made to produce validated questionnaires that capture the time course of exacerbations, well-being, and fatigue in patients with COPD [15–17], similar attention has not been paid to the occurrence of symptoms at night or early in the morning. This study used data from a simple, non-linear, scoring system that provided a semi-quantitative assessment of whether COPD symptoms were present at night. There was evidence of night-to-night variation in symptomatology, with some nights being worse than others. Hence, to capture events throughout the study, data were averaged over 7-day periods and patients who did and did not experience awakenings on at least one night due to respiratory symptoms were identified. During the baseline run-in phase (Week 0), half of the patients experienced at least one night with respiratory symptoms that caused night-time awakening. Night-time awakening score was correlated with the use of rescue medication during the baseline period and overall (Weeks 1–13), with correlation coefficients in the range 0.3–0.5. This moderate correlation reflects, in part, the use of rescue therapy for other reasons and the fact that, although symptoms may awaken a patient, they are not always severe enough for them to seek inhaled treatment. Nonetheless, the correlation between rescue therapy and this important clinical symptom of COPD, suggests that this relatively easily monitored variable may be useful in evaluating how well the disease is controlled, as is commonly used for assessment of asthma control [18]. In the placebo group, the night-time awakening scores were similar at baseline through to Week 13 indicating a consistency in the data, and suggesting that patients who report symptoms at night are likely to find this a persistent problem.

Tiotropium is an effective long-acting inhaled bronchodilator with duration of action of more than 24 h; this effect is supported in this study by the observed improvement in morning PEFR. Tiotropium’s long duration of action is also supported by its effect on night-time awakening. Although during the baseline period, the likelihood of patients reporting night-time awakenings was similar in both groups, by Week 13 there was a significant reduction in these events in patients treated with tiotropium as compared with placebo (adjusted overall treatment means 0.356 and 0.421, respectively; *p* < 0.001). This difference achieved statistical significance, but its clinical importance is questionable. However, the data are the mean of scores from many patients whose night-time awakenings were relatively infrequent (see below). The treatment effect was evident in those who had reported night-time awakenings during the run-in period and were experiencing the symptoms of interest when randomized to treatment. The improvement in night-time symptoms in the tiotropium-treated group was paralleled by reduced rescue medication use, which was seen throughout the 24-h day. Although rescue medication use was lowest between midnight and 6 am, there were still significantly fewer doses taken by patients receiving tiotropium compared with those receiving placebo over this time period (significantly fewer for seven out of 13 weeks and for the overall Weeks 1–13 mean). There were also positive effects on health status and overall levels of fatigue from the intervention, but how much of this could be attributed to the decrease in night-time symptoms is hard to assess. Improvements in early morning symptoms have been reported with other bronchodilator combinations and with other long-acting inhaled bronchodilators [19], although these changes have not always been confirmed when assessed in larger patient groups [20]. Nevertheless, it seems likely that an improvement in lung mechanics overnight will either suppress the production of symptoms or possibly increase the threshold of lung function impairment that needs to be achieved to trigger an awakening. At present there are insufficient data to know which mechanisms are more important. Other studies looking at the effect of tiotropium on awakenings have failed to show an effect, likely due to limitations in study design [21]. This emphasizes the difficulty of applying existing questionnaire methodology where random differences in initial night-time awakenings can occur between treatment groups, as was the case with the Beier et al. study [21].

In this study of patients with moderate-to-severe COPD, only half the patients reported night-time awakenings at baseline due to COPD symptoms. This is in line with other studies, where the percentage of baseline night-time awakenings varied between 35 % and 57 % of patients [22, 23]. However, this may underestimate the true impact of night-time symptoms, as a cross-sectional European survey of 2807 patients found that 78 % of patients with COPD reported night-time sleep disturbances; a problem that worsened with disease severity [24]. Clearly, effective control of COPD-related symptoms is needed across the 24-h day.

This post hoc analysis has a number of limitations. In this study, the majority of patients had only a limited number of night-time awakenings at baseline, which may have reduced the ability to show strong treatment effects. In addition, the clinical impact of the findings is unknown, as the minimal clinically important difference for night-time awakening is not established and warrants further investigation. The studies reported here were undertaken in 1997, at a time when background medications, such as inhaled LABAs and corticosteroids, were less commonly used than is now the case. While this allows us to explore the impact of sustained bronchodilation on night-time symptoms and rescue bronchodilator use, it may be less representative of current therapy, which may include other long-acting bronchodilators alone or in combination. In this study, LABAs were not permitted for at least 1 month prior to the beginning of the baseline period or throughout the study period and inhaled corticosteroids were permitted if at a stable dose for at least 6 weeks prior to screening and throughout the study period. Furthermore, as noted, the metrics for night-time awakenings were relatively crude and did not take account of individual night-time variability; this is an area that merits further exploration. Likewise, the development of more specific, validated questionnaires better able to quantify the impact of night-time symptoms would be useful. Finally, it would help to have data from a healthy, age-matched control population to understand the influence of age-related impairment on sleep quality that is not COPD related. However, in this study, patients were asked to only consider awakenings that they felt were triggered by their respiratory symptoms. Despite these limitations, we believe that our data represent the impact of sustained bronchodilation in patients with COPD not selected on the basis of prior nocturnal symptoms and hence are applicable to ‘real-world’ patients.

## Conclusions

In conclusion, it was found that significant nocturnal awakenings correlated with COPD symptoms, and were present at least once weekly in half of the study patients during the baseline period. These events likely contribute to overall health status impairment. While it cannot be stated with certainty that amelioration of these night-time events positively affects health status, experience in asthma suggests that this may be the case. Encouragingly, morning-dosed tiotropium appears to decrease night-time symptoms and reduce the need for rescue treatment. However, these data also indicate that the problem is not abolished, and so further studies are required to explore the etiology of night-time awakening in this patient population, and whether different or more intensive treatments can favorably impact this potentially troublesome problem.

## Additional files

Additional file 1: Table S1. Energy and fatigue questionnaire. Patients were asked to choose one option in each question that best described how they felt. (PDF 12 kb) Additional file 2: Figure S1. Adjusted weekly mean rescue medication doses/24 h. A dose of rescue medication use was defined as one or two puffs of albuterol. Data are mean ± standard error. The means are adjusted for center effects and baseline. The overall mean is the average over the 13 weekly means. ****p* < 0.001 for tiotropium versus placebo. (PDF 82 kb) Additional file 3: Figure S2. Thirty six Item Short-Form Health Survey (SF-36) assessed at baseline and Week 13 (Day 92). Data are adjusted mean ± standard error. The means are adjusted for center effects and baseline. A score of zero indicates worst health and a score of 100 indicates best health. **p* < 0.05, ***p* < 0.01 tiotropium versus placebo at Week 13. (PDF 389 kb) Additional file 4: Table S2. Pearson’s correlation coefficients: night-time awakening scores and 36-item Short-Form Health Survey (SF-36) scores at baseline, Week 13, and overall (Weeks 1–13). (PDF 212 kb) Additional file 5: Energy–fatigue questionnaire results. **Table S3** Adjusted mean energy–fatigue questionnaire scores assessed at baseline and Week 13 (Day 92). **Table S4** Pearson’s correlation coefficients: night-time awakening scores and energy–fatigue questionnaire data. (PDF 207 kb)

## Abbreviations

COPD: Chronic obstructive pulmonary disease
ECSC: European Community for Steel and Coal
FEV_1_: Forced expiratory volume in 1 s
FVC: Forced vital capacity
HRQoL: Health-related quality-of-life
LABA: Long-acting beta-agonist
PEFR: Peak expiratory flow rate
SE: Standard error
SF-36: 36-Item Short-Form health survey
