# Supplementary material for: Effect of tiotropium on night-time awakening and daily rescue medication use in patients with COPD
Source: Respir Res. 2016 Mar 12;17:27. doi: 10.1186/s12931-016-0340-9 (PMC4789269; doi:10.1186/s12931-016-0340-9)
Supplement: Additional file 1: Table S1. — Energy and fatigue questionnaire. Patients were asked to choose one option in each question that best described how they felt. (PDF 12 kb) [file 12931_2016_340_MOESM1_ESM.pdf]

Table S1 Energy and fatigue questionnaire. Patients were asked to choose one option in each question that best described how they felt

How would you rate your energy level these days?
1 = Very good
2 = Good
3 = Fair
4 = Poor
5 = Very poor
How would you rate your level of fatigue these days?
1 = Very severe
2 = Severe
3 = Moderate
4 = Mild
5 = Very mild
6 = No fatigue
How would you rate the severity of your respiratory condition these days?
1 = Very severe
2 = Severe
3 = Moderate
4 = Mild
5 = Very mild
6 = No problem at all
