# Supplementary material for: Effect of tiotropium on night-time awakening and daily rescue medication use in patients with COPD
Source: Respir Res. 2016 Mar 12;17:27. doi: 10.1186/s12931-016-0340-9 (PMC4789269; doi:10.1186/s12931-016-0340-9)

Figure S1. Adjusted weekly mean rescue medication doses/24 hours. A dose of rescue medication use was defined as one or two puffs of albuterol. Data are mean ± standard error. The means are adjusted for center effects and baseline. The overall mean is the average over the 13 weekly means. ***p < 0.001 for tiotropium versus placebo.

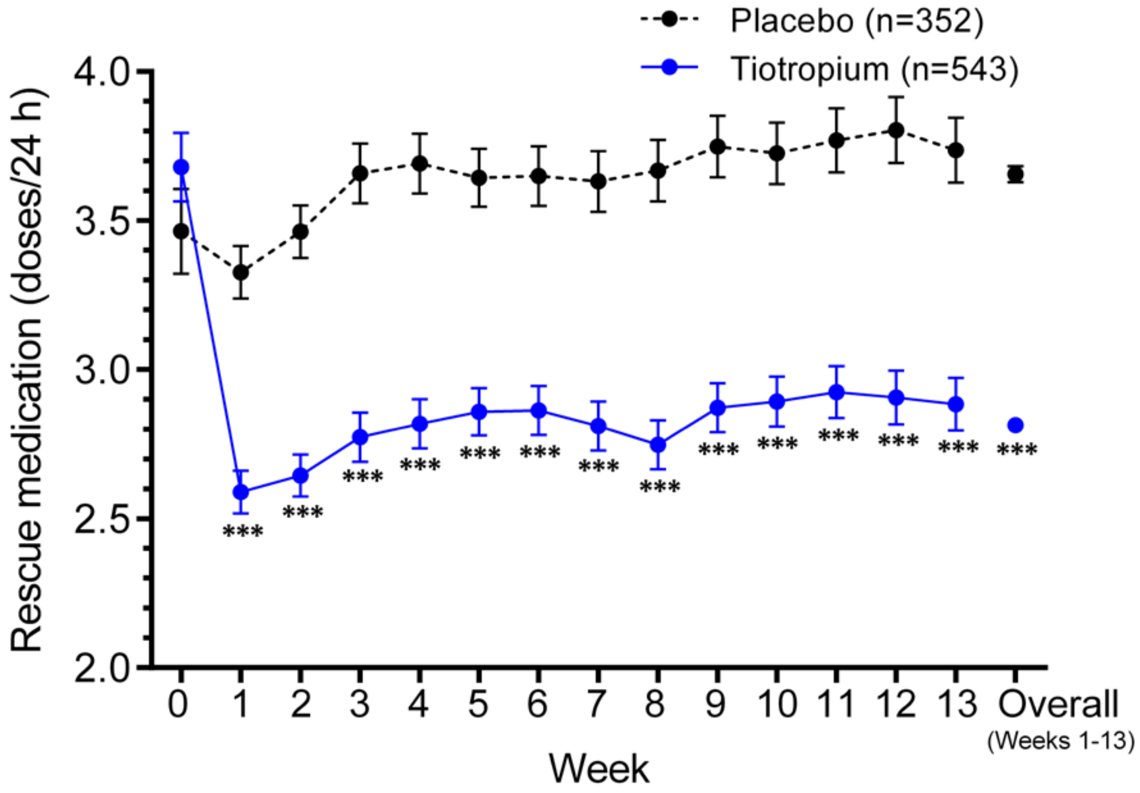

Supplement: Additional file 2: Figure S1. — Adjusted weekly mean rescue medication doses/24 h. A dose of rescue medication use was defined as one or two puffs of albuterol. Data are mean ± standard error. The means are adjusted for center effects and baseline. The overall mean is the average over the 13 weekly means. ***p < 0.001 for tiotropium versus placebo. (PDF 82 kb) [file 12931_2016_340_MOESM2_ESM.pdf]
