# Supplementary material for: Effect of tiotropium on night-time awakening and daily rescue medication use in patients with COPD
Source: Respir Res. 2016 Mar 12;17:27. doi: 10.1186/s12931-016-0340-9 (PMC4789269; doi:10.1186/s12931-016-0340-9)

**Figure S2 36-Item Short-Form Health Survey (SF-36) assessed at baseline and Week 13 (Day 92).** Data are adjusted mean ± standard error. The means are adjusted for center effects and baseline. A score of zero indicates worst health and a score of 100 indicates best health. *p < 0.05, **p < 0.01 tiotropium versus placebo at Week 13.

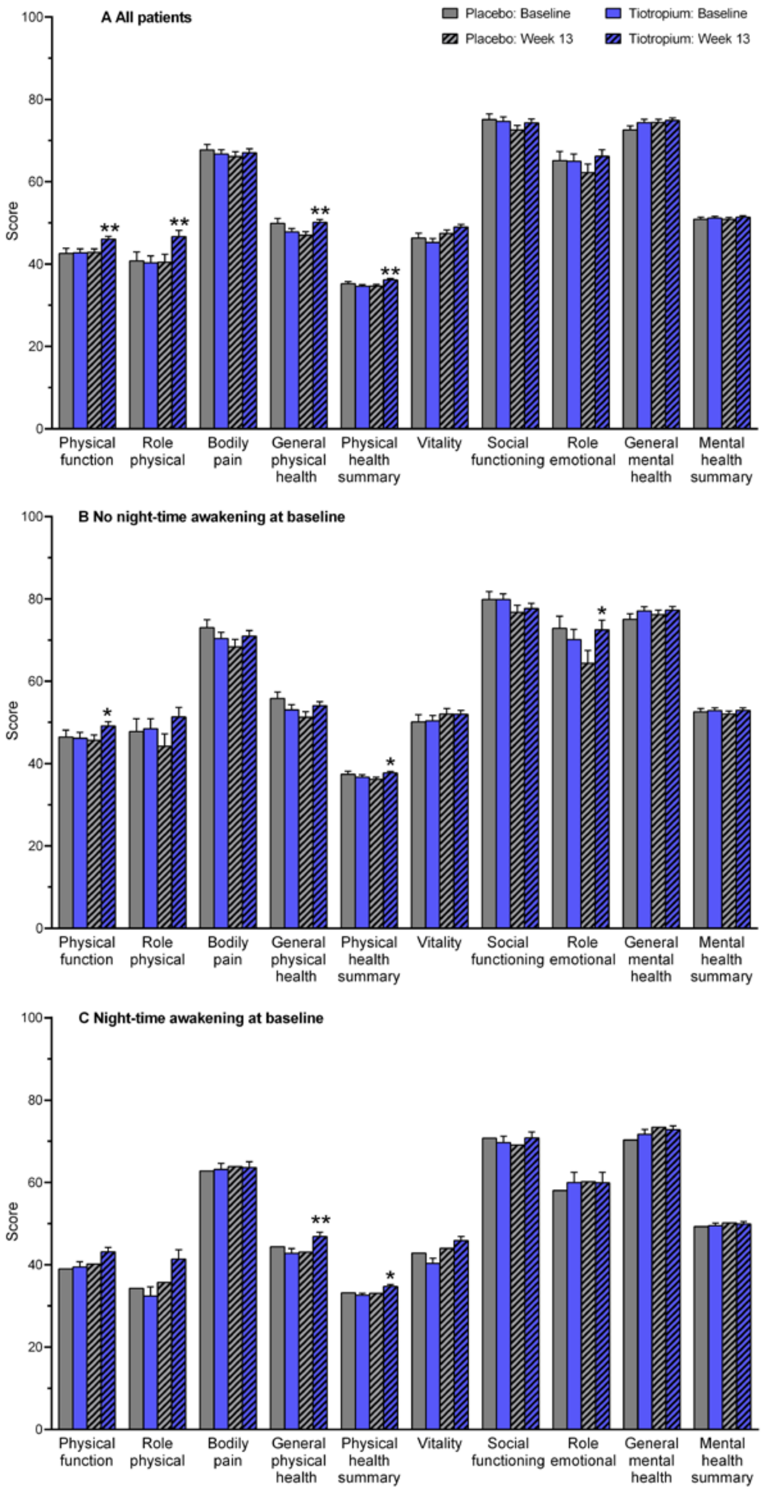

Supplement: Additional file 3: Figure S2. — Thirty six Item Short-Form Health Survey (SF-36) assessed at baseline and Week 13 (Day 92). Data are adjusted mean ± standard error. The means are adjusted for center effects and baseline. A score of zero indicates worst health and a score of 100 indicates best health. *p < 0.05, **p < 0.01 tiotropium versus placebo at Week 13. (PDF 389 kb) [file 12931_2016_340_MOESM3_ESM.pdf]
