# Supplementary material for: Effect of tiotropium on night-time awakening and daily rescue medication use in patients with COPD
Source: Respir Res. 2016 Mar 12;17:27. doi: 10.1186/s12931-016-0340-9 (PMC4789269; doi:10.1186/s12931-016-0340-9)
Supplement: Additional file 4: Table S2. — Pearson’s correlation coefficients: night-time awakening scores and 36-item Short-Form Health Survey (SF-36) scores at baseline, Week 13, and overall (Weeks 1–13). (PDF 212 kb) [file 12931_2016_340_MOESM4_ESM.pdf]

Table S2 Pearson's correlation coefficients: night-time awakening scores and 36-item Short-Form Health Survey (SF-36) scores at baseline, Week 13, and overall (Weeks 1–13)

Correlation with night-time awakening[a,b]		Tiotropium		Placebo	
		n	r	n	r
Physical Function	Baseline	520	–0.225***	328	–0.286***
	Week 13	520	–0.175***	328	–0.285***
	Overall (Weeks 1–13)	520	–0.216***	328	–0.269***
Role Physical	Baseline	519	–0.216***	328	–0.292***
	Week 13	519	–0.181***	328	–0.298***
	Overall (Weeks 1–13)	519	–0.190***	328	–0.226***
Bodily Pain	Baseline	520	–0.258***	328	–0.223***
	Week 13	520	–0.178***	328	–0.215***
	Overall (Weeks 1–13)	520	–0.193***	328	–0.207***
General Physical Health	Baseline	520	–0.281***	327	–0.326***
	Week 13	520	–0.194***	327	–0.302***
	Overall (Weeks 1–13)	520	–0.210***	327	–0.296***
Physical Health Summary[c]	Baseline	517	–0.260***	325	–0.320***
	Week 13	517	–0.178***	325	–0.309***
	Overall (Weeks 1–13)	517	–0.209***	325	–0.284***
Vitality	Baseline	518	–0.297***	327	–0.278***
	Week 13	518	–0.183***	327	–0.308***
	Overall (Weeks 1–13)	518	–0.194***	327	–0.265***
Social Functioning	Baseline	520	–0.295***	328	–0.237***
	Week 13	520	–0.233***	328	–0.380***
	Overall (Weeks 1–13)	520	–0.257***	328	–0.329***
Role Emotional	Baseline	520	–0.217***	327	–0.213***
	Week 13	520	–0.208***	327	–0.277***
	Overall (Weeks 1–13)	520	–0.227***	327	–0.206***
General Mental Health	Baseline	518	–0.285***	327	–0.216***
	Week 13	518	–0.221***	327	–0.208***
	Overall (Weeks 1–13)	518	–0.235***	327	–0.198***
Mental Health Summary[d]	Baseline	517	–0.278***	325	–0.217***
	Week 13	517	–0.233***	325	–0.281***
	Overall (Weeks 1–13)	517	–0.238***	325	–0.230***

***$p < 0.001$.
[a]SF-36 domain scores for Week 13 were measured on Day 92.
[b]Overall night-time awakening (overall mean of Weeks 1–13 weekly means) versus SF-36 domain scores at Week 13 (Day 92).
[c]Summary of Physical Functioning, Role Physical, Bodily Pain, General Physical Health.
[d]Summary of Vitality, Social Functioning, Role Emotional and General Mental Health.
