# Supplementary material for: Effect of tiotropium on night-time awakening and daily rescue medication use in patients with COPD
Source: Respir Res. 2016 Mar 12;17:27. doi: 10.1186/s12931-016-0340-9 (PMC4789269; doi:10.1186/s12931-016-0340-9)
Supplement: Additional file 5: — Energy–fatigue questionnaire results. Table S3 Adjusted mean energy–fatigue questionnaire scores assessed at baseline and Week 13 (Day 92). Table S4 Pearson’s correlation coefficients: night-time awakening scores and energy–fatigue questionnaire data. (PDF 207 kb) [file 12931_2016_340_MOESM5_ESM.pdf]

Energy–fatigue questionnaire results

At Week 13, energy and severity of condition scores were slightly more favorable for the tiotropium treatment group compared with the placebo group ($p < 0.05$; Table S4); these small differences are of uncertain clinical significance.

For both tiotropium and placebo, at baseline, Week 13 and overall (Weeks 1–13), fatigue and severity of condition scores were negatively correlated with night-time awakening scores, while energy score was positively correlated with night-time awakening scores (all $p<0.001$; Table S5).

Table S3 Adjusted mean energy–fatigue questionnaire scores assessed at baseline and Week 13 (Day 92)

		Tiotropium		Placebo	
		n	Mean ± SE	n	Mean ± SE
Energy	Baseline	533	2.972 ± 0.037	335	2.949 ± 0.048
	Week 13	533	2.802 ± 0.031*	335	2.929 ± 0.040
Fatigue	Baseline	532	3.346 ± 0.042	335	3.325 ± 0.055
	Week 13	532	3.415 ± 0.037	335	3.359 ± 0.046
Severity of condition	Baseline	531	2.957± 0.035	335	2.899 ± 0.047
	Week 13	531	3.166 ± 0.033*	335	3.046 ± 0.042

*$p < 0.05$ for tiotropium versus placebo.

Data are mean ± standard error.

The means are adjusted for center effects and baseline.

Energy: 1= very good; 2= good; 3 = fair; 4 = poor; and 5= very poor.

Fatigue: 1 = very severe; 2 = severe; 3 = moderate; 4 = mild; 5 = very mild; and 6 = no fatigue.

Severity of (respiratory) condition: 1 = very severe; 2 = severe; 3 = moderate; 4 = mild; 5 = very mild; and 6 = no problems at all.

Table S4. Pearson's correlation coefficients: night-time awakening scores and energy–fatigue questionnaire data

Correlation with night-time awakening[a]		Tiotropium		Placebo	
		n	r	n	r
Energy	Baseline	533	0.270***	335	0.300***
	Week 13	533	0.203***	335	0.355***
	Overall (Weeks 1–13)	533	0.217***	335	0.320***
Fatigue	Baseline	532	–0.183***	335	–0.260***
	Week 13	532	–0.174***	335	–0.265***
	Overall (Weeks 1–13)	532	–0.213***	335	–0.242***
Severity of condition	Baseline	531	–0.180***	335	–0.282***
	Week 13	531	–0.175***	335	–0.198***
	Overall (Weeks 1–13)	531	–0.190***	335	–0.209***

***$p < 0.001$.

[a]Health-related quality-of-life measures at Week 13 were measured on Day 92.
